# Evaluation of *Crocus sativus* L. Stigma Phenolic and Flavonoid Compounds and Its Antioxidant Activity 

**DOI:** 10.3390/molecules15096244

**Published:** 2010-09-06

**Authors:** Ehsan Karimi, Ehsan Oskoueian, Rudi Hendra, Hawa Z.E. Jaafar

**Affiliations:** 1 Department of Crop Science, Faculty of Agriculture, Universiti Putra Malaysia (UPM), 43400 UPM Serdang, Selangor, Malaysia; 2 Department of Microbiology, Faculty of Biotechnology and Biomolecular Sciences, Universiti Putra Malaysia (UPM), 43400 UPM Serdang, Selangor, Malaysia; 3 Department of Biochemistry, Faculty of Biotechnology and Biomolecular Sciences, Universiti Putra Malaysia (UPM), 43400 UPM Serdang, Selangor, Malaysia

**Keywords:** saffron, DPPH free radical scavenging activity, ferric reducing antioxidant power, reversed-phase HPLC, secondary metabolites

## Abstract

Saffron (*Crocus sativus* L.) belongs to the Iridaceae family. The stigma of saffron has been widely used as spice, medicinal plant, and food additive in the Mediterranean and Subtropical countries. Recently, attention has been paid to the identification of new sources of safe natural antioxidants for the food industry. The antioxidant activities of spices are mainly attributed to their phenolic and flavonoid compounds. Saffron is one of the spices believed to possess antioxidant properties, but information on its antioxidant activity and phenolic, flavonoids compound are rather limited, therefore this research was carried out to evaluate the antioxidant activity of saffron stigmas extracted with different solvents. The phenolic and flavonoid compounds of saffron were also examined using reversed phase (RP)-HPLC. Results showed that saffron stigma possess antioxidant activity. The free radical scavenging and ferric reducing power activities were higher for the methanolic extract of saffron stigma at a concentration of 300 μg/mL, with values of 68.2% and 78.9%, respectively, as compared to the corresponding boiling water and ethanolic extracts, but the activities were lower than those of antioxidant standards such as BHT and α-tocopherol. The obtained total phenolics value for methanolic saffron extract was 6.54 ± 0.02 mg gallic acid equivalent (GAE)/g dry weight (DW), and for total flavonoids, 5.88 ± 0.12 mg rutin equivalent/g DW, which were also higher than values obtained from the ethanolic and boiling water extracts. In addition, the RP-HPLC analyses indicated the presence of gallic acid and pyrogallol as two bioactive compounds. In summary, saffron stigmas showed antioxidant activity and methanol appeared to be the best solvent to extract the active components, among which the presence of gallic acid and pyrogallol might contribute towards the stigma’s antioxidant properties. Hence, saffron stigma could be applied as a natural antioxidant source for industrial purposes.

## 1. Introduction

In the human body, oxidant–antioxidant imbalance impairs cell functions and immunity, and promotes cell death and DNA damage, which can cause mutations and ultimately contribute towards the development of chronic diseases such as cancer [1,2]. Food composition and food additives play major role in providing the required antioxidants for the body. Although traditionally spices have been used in food preparations to improve the flavor and taste, today they are also frequently used as antioxidant-food supplements. Spices are reported to contain bioactive compounds imparting antioxidant, preservative and antimicrobial properties to the food. Several researches have shown that spices containing phenolic and flavonoid compounds indicated antioxidant activities [3,4,5]. A positive linear correlation among phenolic compounds and flavonoids with antioxidant capacity of spices has also been reported [6]. 

Synthetic antioxidants such as butylated hydroxytoluene (BHT) and butylated hydroxyanisole (BHA) have been widely used for many years to retard lipid oxidation. However, the safety of using these synthetic antioxidants in food industry has become a concern among scientists and leading to current interest in uncovering natural antioxidants. As a result many spices have been evaluated for their antioxidative properties for use as a source in foods. The antioxidants constitute a range of substances that play a role in protecting biological systems against the deleterious effects of oxidative processes on macromolecules, such as proteins, lipids, carbohydrates, and DNA [7]. Many of those substances are plant-derived natural molecules that contribute to the prevention and treatment of diseases in which reactive oxygen species are involved. This protection can be explained by the plant antioxidants’ capacity to scavenge free radicals [8,9]. The importance of antioxidants in maintaining health and protection from coronary heart disease and cancer is of great interest among scientists, food manufacturers and consumers [10,11], which should further intensify the interest in revealing the antioxidant properties of spice or herb plants. 

Saffron, the dried stigmas of a flower scientifically identified as *Crocus sativus *L. has also been identified as a spice with beneficial traits. The origin of saffron is unknown, but it is believed to be originated from the regions of Iran, Turkey and Greece. Recently, saffron has also been cultivated successfully in other areas such as in Europe [12]. Saffron is mostly used as a spice and food colorant and, less extensively, as a textile dye or perfumery ingredient. It is also used in herbal folk medicine for the treatment of numerous illnesses due to its analgesic and sedative properties [13,14,15]. Chemical analyses of saffron extracts by Fernandez [16] revealed the main constituents of the plant to be carotenoids, glycosides, monoterpenes, aldehydes, picrocrocin and antocyanins, flavonoids, vitamins (especially riboflavin and thiamine), amino acids, proteins, starch, mineral matter, and gums; other chemical compounds have also been reported in saffron, but only scanty information on the antioxidant activity of this spice has been documented. Therefore, the research reported herein was carried out to evaluate the presence of phenolic and flavonoid compounds by RP-HPLC as well as assessing the antioxidant activity of saffron stigmas as one of the common food spices. 

## 2. Results and Discussion

### 2.1. Phenolic, flavonoid and antioxidant activities

Results on the phenolic and flavonoid contents of saffron stigma extracts obtained using different solvents are presented in Table 1. Saffron stigmas contained phenolic and flavonoid compounds and diifferent solvents showed different contents of total phenolics and flavonoids. Significant differences (p < 0.05) in the phenolic content of methanolic, ethanolic and boiling water extracts were observed, with values of 6.54, 6.35, and 5.70 mg GAE/g DW, respectively. Similarly, the flavonoid contents were markedly higher in the methanolic extract, with a value of 5.88 mg rutin equivalent/g DW compared to the boiling water extract at 3.86 mg and the ethanolic extract with a value of 2.91 mg rutin equivalent/g DW. 

**Table 1:** Total phenolic and flavonoid content of saffron stigma.

Solvent	Phenolic Content^1^	Flavonoid Content^2^
Ethanol	6.3 ± 0.01^b^	2.9 ± 0.02^c^
Water	5.7 ± 0.04^c^	3.8 ± 0.09^b^
Methanol	6.5 ± 0.02^a^	5.8 ± 0.12^a^

^1^ mg gallic acid equivalent/g DW; ^2^ mg rutin equivalent/g DW; Means with the different letters are significantly different; Values are means of three replications.

**Figure 1:**
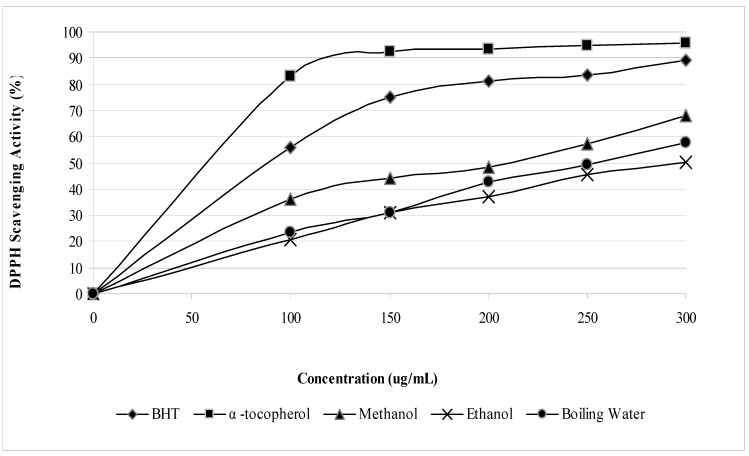
Free radical scavenging activity of *Crocus sativus* extracts and reference antioxidants.

**Figure 2:**
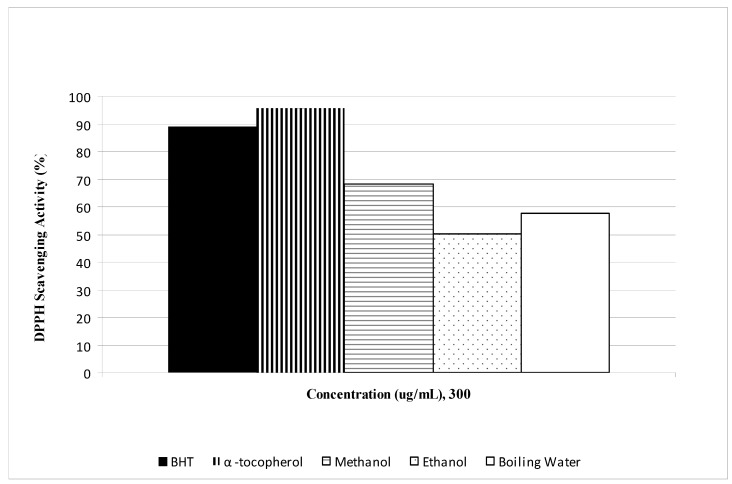
The free radical scavenging activity of *C. sativus* L. extracts using different solvents and reference antioxidants at 300 μg/mL.

DPPH and FRAP assay results revealed the antioxidant activities of saffron stigmas; however, the antioxidant activity was affected by the nature of the solvent used. The free radical scavenging activity (DPPH) assay indicated a steady increase in the free radical scavenging activity by all the extracts and standards in the range of 0 to 300 μg/mL (Figure 1). Free radical scavenging activity of methanolic saffron stigma extract was stronger than that of the boiling water extract, followed by the ethanol one. All the values, however, were lower than those obtained for BHT and α-tocopherol that were used as antioxidant standards. At a concentration of 300 µg/mL (Figure 2) the scavenging activities of saffron extracts and standards on free radicals decreased in the order of α-tocopherol > BHT > methanol > boiling water > ethanol with values of 95.6%, 89.0%, 68.2%, 57.6%, and 50.1%, respectively. The IC_50_ (concentration required to inhibit 50% of DPPH radicals) of α-tocopherol, BHT, and methanol, boiling water and ethanol extract were found to be 89.77, 60.39, 210.79, 255.44, and 299.44 µg/mL, respectively.

**Figure 3:**
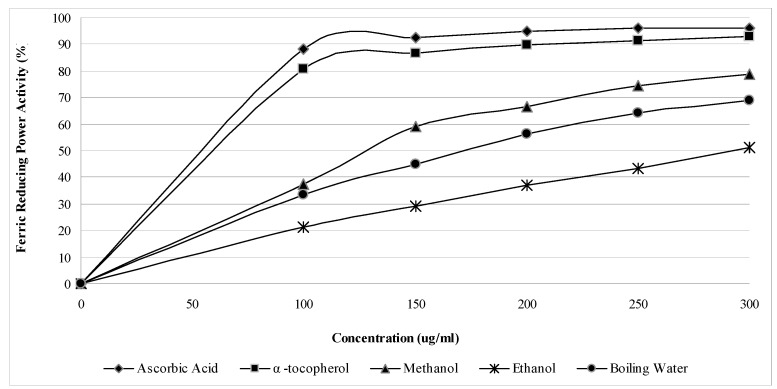
Ferric reducing activity power of *C. sativus* L. stigma extracts and reference antioxidants.

**Figure 4:**
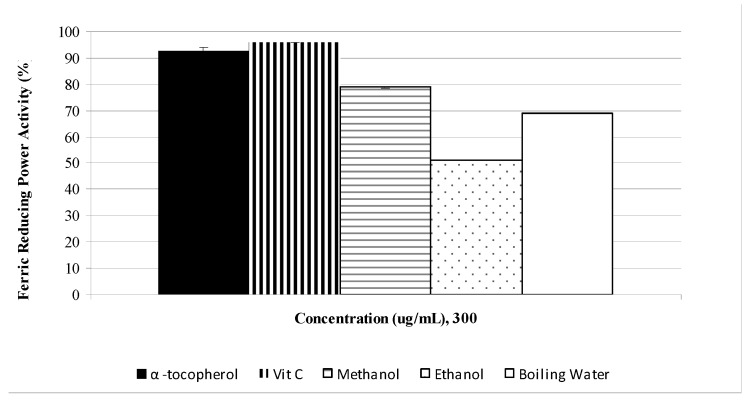
The reductive potential of *C. sativus* L. stigma extracts and reference antioxidants at 300 μg/mL.

The ferric reducing power activity (FRAP) assay was used to determine the reduction potential (Fe^3+^ → Fe^2+^) of saffron stigmas. Similar to the DPPH results, the reductive potential of saffron increased in a dose dependent manner (Figure 3). Saffron appeared to be active in the reduction of Fe^3+^, indicating its antioxidant activity. The ferric reducing power activity of saffron stigmas varied among the extracts, but the values were all lower than those of the standards. Methanolic extract showed a higher reductive potential than the boiling water and ethanolic extracts. The reductive potential of saffron extracts and standards at a concentration of 300 µg/mL (Figure 4) were found to be in the ascending order BHT > α-tocopherol > methanol > boiling water > ethanol, with respective values of 96.1%, 92.9%, 78.9%, 68.7% and 51.3%. 

Phenolic and flavonoid compounds, which are widely found as secondary metabolites in plants, are important due to their ability to serve as antioxidants [17]. Many phenolic compounds have been reported to possess potent antioxidant activity and anti-cancer, anti-carcinogenic, anti-bacterial, anti-viral or anti-inflammatory activities in a greater or lesser extent [18,19,20,21]. Flavonoids, which are found commonly in the leaves, flowering tissues and pollens [22] are an important part of the diet because of their effects on human nutrition [23]. The most important function of flavonoids is their antioxidant activity, as they have been shown to be highly effective scavengers of most types of oxidizing molecules, including singlet oxygen and various free radicals [24,25].

The findings on this work show that extracts of saffron stigmas obtained from the Iranian region possess antioxidantive potential, and are in agreement with data collected by Assimopoulou *et al.* [26] who reported the antioxidant activity of saffron. They belived that the antioxidant activity of saffron could be attributed to two bioactive compounds, crocin and safranal, and a DPPH radical scavenging test on crocin and saffranal exhibited antioxidant activities of 65% and 34%, respectively, at 500 ppm. In this study, the antioxidant activity of the methanolic extract at 300 μg/mL was 68.23%. Chen *et al.* [27], when comparing the antioxidant activity of saffron obtained from China with *Gardenia jasminoides,* reported that the ethanolic extract of Chinese saffron exhibited lower scavenging properties against DPPH radicals, with a value of 107 mg α-tocopherol/g as compared to a *Gardenia *ethanolic extract with a value of 421 mg α-tocopherol/g. Our saffron samples from Khorasan Province (Iran) thus appeared to possess higher antioxidant activity compared to the saffron samples investigated by Assimopoulou *et al. *and Chen *et al.* [26,27]. In the current work, the antioxidant activity in saffron stigma might be attributed to the presence and synergistic effects of phenolic and flavonoid compounds (Table 2 and Table 3), besides any other active compounds present. However, since a hydrolytic extraction was applied, the linkages between flavonoids and phenolics with their corresponding glycosides could probably have broken down, which would lead to an increase in the antioxidant activity of the sample. Referring to Von Gadow *et al.* [28], the presence of glycosides in the flavonoids decreased the antioxidant activity by affecting the donation of hydrogen. The antioxidant capacities of saffron in human plasma were also analyzed by Chatterjee *et al.* [29] who confirmed the antioxidant capacities of saffron using the oxidative stress, nitric oxide and DPPH radical scavenging assays.

**Table 2:** Phenolic compounds in methanolic extract of *C. sativus* L. stigmas.

Phenolic contents (mg/g dry sample)				
Gallic acid	Salicylic acid	Caffeic acid	Vanillic acid	Syringic acid
1.82 ± 0.02	ND	ND	ND	ND

ND: not detected.

**Table 3:** Flavonoid compounds in methanolic extract of *C. sativus* L. stigmas.

Flavonoid contents (mg/g dry sample)						
Apigenin	Kaempferol	Myricetin	Naringin	Quercetin	Pyrogallol	Rutin
ND	ND	ND	ND	ND	1.4 ± 0.05	ND

ND: not detected.

Yen and Duh and Siddhuraju *et al.* [30,31] have reported that the ferric reducing power of bioactive compounds was associated with antioxidant activity. The ferric reducing antioxidant power of the methanolic extract showed higher activity compared to that obtained with the other solvents. This might be due to the presence of higher total phenolics and flavonoids, which play a major role in reducing power activity. Siddhuraju and Becker [32] noted that the plants with higher levels of total phenolics and flavonoids exhibited greater reducing power activity. The presence of different antioxidant activities in the different extracts was associated with the total phenolic, and especially the flavonoid contents, although both contents were affected by the nature of the solvents used (Table 1) as a solvent can be hydrophilic or lipophilic, and thus able to separate different compounds. Thus, the results of the current study showed that the ethanolic extracts appeared to be less effective in reducing power activity and DPPH free radical scavenging activity, which might be explained by the lower total flavonoid content of the ethanolic extract, so the results correlated well with the contents of phenolics and particularly flavonoids.

Shan *et al.* [33] suggested that some of the variations in antioxidant capacity encountered in different studies might also be attributed to genotypic and environmental differences within species, the parts of the plants studied, the time of year the samples were taken (especially for fresh products), and the analytical methods used.

When comparing with other common feed additives and spices, Gulcin *et al.* reported that total phenolic compounds of clove (*Eugenia caryophylate *Thunb.) buds and lavender (*Lavandula stoechas* L) from ethanolic extract were 0.26 mg and 0.22 mg gallic acid equivalent/g DW, respectively. The total phenolic compounds of the ethanolic extract of saffron in the present work was higher (6.35 mg gallic acid equivalent/g DW) than both clove and lavender, but it was lower compared to total phenolic of *Curcuma domestica* and *Curcuma longa* L. (22.9 and 35.6 mg gallic acid equivalent/g DW, respectively), as reported by Chen *et al.* [27]. Saffron also contained higher total flavonoids compared to ethanolic leaf extracts of *Piper betel* (0.664 mg rutin equivalent/g DW) [34]. 

### 2.2. Determination of phenolic and flavonoid compounds by HPLC

**Figure 5:**
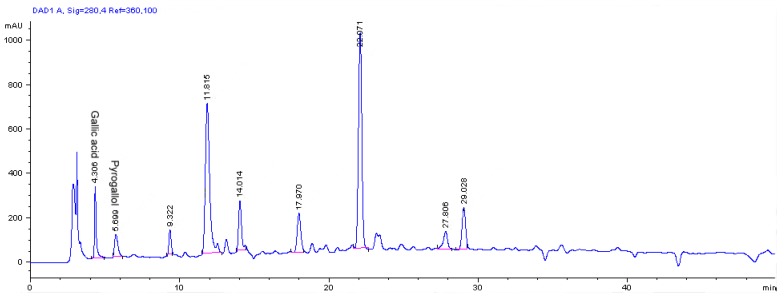
The RP-HPLC chromatogram of phenolic and flavonoid compounds in methanolic extract of *C. sativus* L. stigmas.

Reversed-phase (RP) liquid chromatography was used to determine the phenolic and flavonoid compounds present in the saffron extract. The phenolic and flavonoid compounds were identified based on their retention times and quantified according to the respective standard calibration curves (Figure 5). 

The HPLC chromatogram of the methanolic saffron extract indicated gallic acid (Table 2) as the major phenolic and pyrogallol as the major flavonoid compound present (Table 3), with values of 1.82 ± 0.02 and 1.4 ± 0.05 mg/g dry sample, respectively. According to the standards used no other phenolic, flavonoid and iso-flavonoids compounds were detected (Table 2 and Table 3). Proestos *et al.* [35] have the concentration of gallic acid found in saffron leaves to be 1.2 ± 0.02 mg/100 dry sample. This amount was markedly lower compared to the gallic acid present in the saffron stigmas of the current work. 

Sroka and Cisowski [36] reported the DPPH radical scavenging activity of gallic acid and pyrogallol. This result was also supported by Ramma *et al.* [37] who revealed that gallic acid and a small amount of naringin were responsible for the antioxidant activity of black tea and suggested that there seemed to be a good correlation among gallic acid content and antioxidant activity. According to the result obtained in this study in spite of the presence of gallic acid and pyrogallol, the antioxidant activity of suffron may not be completely attributed by them since the presence of other active compounds contributing to antioxidant activity such as β-carotene, lycopene, vitamin E, ascorbic acid and other organic acids have been reported previously [38,39,40,41].

## 3. Experimental

### 3.1. Plant material

Saffron (*Crocus sativus *L.) used in this study was freshly harvested from a farm located in the city of Kashmar, Khorasan Province, Republic of Iran in October 2008. The stigmas of *C. sativus *L. were separated from the flowers, air-dried for five days, and ground to powder using a pestle and mortar. The ground powder was kept in a dark bottle at -20 ºC for further analyses.

### 3.2. Preparation of saffron extracts

Stigmas of *C. sativus *L. were extracted using three different solvents: ethanol, methanol and boiling water. For methanol and ethanol extraction the method of Crozier *et al.* [42] was followed. An air-dried sample (0.5 g) was weighed and placed in a 100 mL conical flask, and 80% (v/v) ethanol or methanol (40 mL) were added, followed by an addition of 6 M HCl (10 mL). The mixture was refluxed for 2 h at 90 ºC and filtered using Whatman No. 1 filter paper (Whatman, U.K.), followed by with evaporation of the filtrate under vacuum using a rotary evaporator (Buchi, Switzerland). The boiling water extraction was conducted after a method by Gulcin *et al.* [43]. Five grams of ground saffron were placed in a glass beaker and mixed with boiling water (100 mL) followed by magnetic stirring for 15 min. The extract was then filtered and evaporated as mentioned above. The dried crude extract was weighed and dissolved in methanol and stored at -20 ºC for further experiments. 

### 3.3. Total phenolic content

The amount of total phenolic compounds in the saffron extract was determined using the Folin-Ciocalteu reagent according to Halici *et al.* [44]. Total phenolic contents results were expressed as milligrams of gallic acid equivalents (GAE) per gram dry weight (DW).

### 3.4. Total flavonoid content

Total flavonoid compound was measured by an aluminum chloride colorimetric assay based on Zhishen *et al. *[45]. Total flavonoid contents of the extracts were expressed as mg rutin equivalent/g dry weight (DW).

### 3.5. Antioxidant activity (DPPH free radical scavenging activity)

The free radical scavenging activities of the extracts were determined as reported by Gulcin *et al.* [43]. All determinations were conducted for three replicates. Lower absorbance values of the reaction mixture indicated higher free radical scavenging activity. The free radical scavenging activities of the tested samples were expressed as percentage of inhibition and were calculated according to the following equation [46]:

Percent (%) inhibition of DPPH activity = [(A_0_ – A_1_) / A_0_)] × 100 %

where A_0_ is the absorbance value of the blank sample or control reaction, and A_1_ is the absorbance value of the test sample. A curve of percent inhibition or percent scavenging effect against samples concentrations was plotted and the concentration of the sample required for 50% inhibition was determined. The value for each of the test sample was presented as the inhibition curve at 50% or IC_50_. 

### 3.6. Ferric reducing antioxidant power (FRAP)

The ferric reducing property of the extracts was determined using an assay described by Yen and Chen [47]. The assay was carried out in triplicate. BHT and α-tocoferol were used as standard antioxidants.

### 3.7. Determination of phenolic and flavonoid compounds by HPLC

The phenolic and flavonoid compounds of saffron were quantitatively measured by a reversed-phase HPLC technique based on the method described by Crozier *et al.* [42] with some modifications. The phenolic compound standards used were gallic acid, syringic acid, vanillic acid, salicylic acid, and caffeic acid, and the flavonoid compound standards were quercetin, rutin, myricetin, kaempferol, naringin, apigenin, genistein, daidzein, and pyrogallol. An aliquot of sample extract was loaded on a Agilent-1200 series high-performance liquid chromatography (HPLC) instrument equipped with a UV-Vis photodiode array (DAD) detector, binary pump, vacuum degasser, auto sampler and an analytical column (Intersil ODS-3 5 μm 4.6 × 150 mm Gl Science Inc). The solvents used were deionized water and acetonitrile, whilst the pH of water was adjusted with trifluoroacetic acid to 2.5. The phenolic and iso-flavonoid compounds were detected at 280 nm, while flavonoid compounds were detected at 350 nm. The column was equilibrated by 85% solvent A (which is it) and 15% solvent B then the ratio of solvent B was increased to 85% in 50 min followed by reducing solvent B to 15% in 55 min. This ratio continued to the 60th min for the next analysis at a flow rate at 0.6 mL/min.

### 3.8. Statistical analysis

The antioxidant activities, total phenolic and flavonoid contents were analyzed using analysis of variance (ANOVA) with Statistical Analysis System (SAS) Version 9 (SAS Institute, Cary, NC). Significant differences among means from triplicate analyses (p < 0.05) were determined by Duncan’s Multiple Range Test.

## 4. Conclusions

Saffron stigmas were found to possess antioxidant activity, hence, saffron is a promising natural product in this respect. Different solvents affected the total phenolic and flavonoid contents of the extracts and led to the observation of different antioxidative efficacy. The gallic acid and pyrogallol as bioactive compounds present in saffron stigma contributed in its antioxidant activity. It is suggested that saffron stigma besides being colorant could play a role as antioxidant source, which might enhance the quality of the products in functional foods, beverages, drinks, pharmaceutical and cosmaceutical industries.
